# Recent Progress in Keloid Mechanism and Treatment: A Comprehensive Review

**DOI:** 10.3390/biomedicines13092276

**Published:** 2025-09-16

**Authors:** Lucia Merlino, Mattia Dominoni, Martina Rita Pano, Marianna Francesca Pasquali, Roberto Senatori, Grazia Zino, Barbara Gardella

**Affiliations:** 1 Department of Medical-Surgical Sciences and Biotechnologies, Sapienza University of Rome, 00161 Rome, Italy; lucia.merlino@uniroma1.it; 2 Department of Clinical, Surgical, Diagnostic and Pediatric Sciences, University of Pavia, 27100 Pavia, Italy; martinarita.pano01@universitadipavia.it (M.R.P.); mariannepasquali@gmail.com (M.F.P.); barbara.gardella@gmail.com (B.G.); 3 Department of Obstetrics and Gynecology, IRCCS Fondazione Policlinico San Matteo, 27100 Pavia, Italy; 4 SICPV, Italian Society of Colposcopy and Cervicovaginal Pathology, 50142 Firenze, Italy; robertosenatori@gmail.com; 5 IDI-IRCCS Istituto Dermatopatico dell’Immacolata, 00167 Rome, Italy; grazia.zino@gmail.com

**Keywords:** keloids, topical treatment, surgery

## Abstract

Keloids are abnormal fibroproliferative responses in the skin that often occur without an apparent injury. Their pathogenesis remains incompletely understood, though genetic, environmental, and biochemical factors are believed to contribute. Topical medications (mostly TCA injection) are the most used treatments followed by surgery, alone or in association with other therapeutic options. In most cases, improvement has been described. A combination of altered collagen synthesis, overactive fibroblasts, and immune response contributes to keloid formation. Genomic studies have identified specific mutations, and the role of growth factors such as TGF-β has been confirmed as a key player in keloid pathogenesis. Although great improvements have been made from the molecular point of view and keloids are more easily diagnosed and treatable nowadays, they remain very challenging, having a great impact on quality of life. Their recurrence is still very high. Understanding genetic predisposition and microenvironmental influences is critical for developing more effective therapies. Advances in molecular research and clinical strategies are improving our understanding of keloids, but further studies are needed to establish precise diagnostic markers and more effective long-term treatments.

## 1. Introduction

Keloids are persistent, progressive, and exaggerated fibroproliferative skin lesions that usually develop after traumas, extending beyond the scar’s margins, with an infrequent tendency to spontaneous regression [1]. Although they are typically brought on by inflammation or dermal injury, they are not scars in the traditional meaning of the word because they expand and develop over decades. People with non-European ancestry, particularly those from sub-Saharan Africa, are more likely to develop keloids due to an autosomal dominant familial predisposition. Keloids have a strong effect on quality of life due to the frequent occurrence of severe neurogenic pain and pruritus, as well as frequent episodes of suppuration, in addition to deformities. The primary basis for diagnosis is a thorough understanding of the clinical features of keloids. They appear as bulging, claw-like plaques or nodules, characterized by a firm consistency and a range of pigmentation, from violaceous to light or deep brown. Specific skin sites are more prone to keloid development, such as on the ear lobes, anterior chest wall, and shoulders [2]; soles, palms, and genitalia are less frequently afflicted [3]. Histopathologically, keloids are characterized by the presence of whorls and nodules of thick, hyalinized collagen bundles or keloidal collagen with mucinous ground substance and relatively few fibroblasts [4]. Keloidogenesis is not fully understood; this abnormal wound healing has been linked to aberrant collagen turnover, alteration in growth factor regulation, immune dysfunction, sebum reaction, genetic predisposition, and hormonal influence [5]. As shown in Table 1, in most cases, multiple keloids have been associated with various diseases, such as atopic conditions and asthma, with some medications and with specific genetic syndromes.

Different therapeutic options are currently available for keloid treatment, which include surgical, non-surgical, and combined treatments [6]. The most common medical approach is represented by intralesional injection of corticosteroids such as triamcinolone acetonide (TAC) alone or in association with 5-fluorouracil. Other injectable drugs are bleomicin, botulinum A, and interferons. Laser devices, such as YAG laser, CO_2_ laser, and KTP (Potassium-Titanyl-Phosphate) laser, have been shown to be effective. New medical approaches, such as intralesional verapamil or UV-A phototherapy, have emerged in recent years.

In this article, we present a comprehensive review of cases present in the literature regarding keloids in male and female populations.

Table 1 reports the main findings derived from the literature data about the correlation between keloids and some etiological/predisposing factors.

Genetic susceptibility, inflammation, mechanical stress, tissue hypoxia, delayed-type hypersensitivity, and metabolic dysfunction are, in fact, some of the variables that contribute to the likely multifactorial etiology of keloids. In particular, there are some reports that show autosomal dominant inheritance in families with varied expression and insufficient clinical penetration.

As for immunological factors, it is known that the healing process derives from a balance between growth factors and cytokines. In keloid formation, there is an increased production of TNF-α, IFN-β, and IL-6 and a diminution in IFN-a, IFN-γ, and TNF-β. Overgrowth and excessive development at the site of damage result from this malfunction, which raises collagen production 20 times higher than a normal scar with an abundance of type III collagen, chondroitin 4-sulfate, and glycosaminoglycan.

Eventually, some comorbidities (e.g., asthma) and some drugs, like isotretinoin, are also known to be related to keloid formation and proliferation, probably because they act on the same molecular pathways that lead to keloid development.

In the literature research on keloids, no meta-analysis is present, and only one randomized clinical trial (RCT) and two systematic reviews about treatment options were found. Only one review from Jfri et al. [7] is available, which provides a summary of the cases with keloids reported in the literature and organized according to their associated medical conditions.

Of the 26 papers that we have analyzed, 7 were case reports and 21 were case series. Given the type of articles present in the literature on keloids, we have a small sample size of the population examined.

In total, 33 patients were described, 16 of which were female and 17 were male between the ages of 7 and 81 years old, with a mean age of 38 years old. Of these, 15 were affected by specific genetic syndromes known to be associated with keloids. A total of 26 presented multiple keloids, which were localized in different body segments but more often in the back, chest, and shoulder areas. In only one case, keloids afflicted the genital area. In solely 18 cases, the proposed treatment was specified: in 7 cases, a surgical approach was chosen, and in 4 cases, radiotherapy followed the surgical excision. When available, histological exams revealed normal keloid characteristics. In 12 cases, a topic treatment was offered, which usually involves triamcinolone injections. In two cases, a worsening of symptoms, including moderate regrowth, was reported, and in five cases, no change in overall conditions was appreciated. In eight cases, an amelioration, even partial, of symptoms has been described.

Two rare cases of keloids in elderly patients were described, one postauricular in a male patient, excised because a carcinoma was suspected, with no recurrences [8], and the other on the anterior mid-chest in a female patient, treated with intralesional injections of corticosteroids [9].

**Table 1:** Main findings from the studies included in the literature search about keloids.

Variables	Main Findings
Genetic factors [9,10,11,12,13,14,15,16,17,18,19,20,21,22,23]	Familial cases of keloids suggest genetic predisposition. The genetic syndromes/pathologies associated with keloids found in the literature were, in order of frequency, as follows: Rubinstein-Taybi syndrome, congenital muscular torticollis, Bethlem myopathy, X-Linked Syndrome, Dubowitz and Noonan syndrome, unilateral cryptorchidism, conjunctivo-corneal dystrophy.
Immunological factors [9,21]	Numerous genotypes linked to immunological pathways have been found, such as polymorphisms of the trans-forming growth factor (TGF)-b receptor and interleukin (IL)-6. This leads to aberrant wound healing. Furthermore, a characteristic of keloid tissue is immune cell infiltration, due to an imbalance between growth factors and cytokines.
Comorbidities [15,24,25,26,27,28,29]	Tuberculosis, hypertension, Behcet’s disease, acne, asthma, chronic idiopathic angioedema, a rare case of nephrogenic systemic fibrosis after renal transplant, Dupuytren’s contracture, diabetes mellitus, osteoporosis, polyfibromatosis, hidradenitis suppurativa.
Drugs [25,30]	Acne treated with isotretinoin. Ductal carcinoma of the breast treated with letrozole.

## 2. Mechanism and Diagnosis

Keloids are benign fibroproliferative lesions that result from an exaggerated wound healing response, characterized by excessive collagen deposition and fibrosis. These lesions can potentially develop in any anatomical region, often without an obvious predisposing trauma, making their study complex and multifactorial. Reports suggest that about 10–15% of all keloid cases do not have a clear precipitating factor [31].

### 2.1. Epidemiology

The prevalence and incidence of keloids are influenced by genetic and environmental factors. Studies have shown that keloid formation is more common in individuals with darker skin types, particularly those of African, Hispanic, and Asian origin. According to a study by Al-Attar et al. [5], the incidence of keloids in individuals of African descent is approximately 5–15%, while in Caucasians, it is less than 1%. This increased susceptibility is believed to be due to genetic factors, as certain loci on chromosomes 2 and 7 have been linked to keloid formation.

They are also more common in females (20.4%) compared to males (12.9%) in the general population [32], and this may be attributed to hormonal differences, skin characteristics, or other genetic factors. Estrogen has been shown to enhance collagen synthesis in fibroblasts, which could predispose women to keloid formation. This is supported by the observation that keloid development is often seen during puberty or pregnancy, periods characterized by increased estrogen levels.

Keloid susceptibility is also influenced by age, with the maximum prevalence occurring between the ages of 10 and 30 [33].

### 2.2. Pathophysiology

The pathophysiology of keloids is a complex interplay of genetic predisposition, aberrant wound healing, and environmental factors. Their formation is rare, and it is still controversial whether it is truly spontaneous, since the scar tissue may develop following a minor injury or inflammatory response that was too unimportant for the person to recall [27].

Under normal conditions, wound healing proceeds through four stages: hemostasis, inflammation, proliferation, and remodeling. In keloid formation, the normal remodeling phase is disrupted, resulting in excessive collagen deposition, particularly type I and III collagen. This fibrotic tissue overgrowth extends beyond the original wound margins, leading to the characteristic raised, hyperpigmented lesion.

Fibroblasts have a pivotal role in the pathogenesis of keloids. These cells may be divided into 4 subpopulations: secretory-papillary, secretory-reticular, mesenchymal, and pro-inflammatory. The mesenchymal subpopulation is the most increased in keloid tissue compared to normal scar tissue. In addition, mesenchymal fibroblasts show an augmented expression of secretory proteins such as POSTN, COMP, COL11A1, ASPN, and COL5A2 [34].

At the molecular level, keloid formation is thought to involve dysregulated fibroblast activity, excessive collagen synthesis, and impaired collagen degradation. Key cytokines such as transforming growth factor-beta (TGF-β), interleukin-6 (IL-6), and platelet-derived growth factor (PDGF) play pivotal roles in fibroblast proliferation and extracellular matrix production. Specifically, TGF-β is critical in the upregulation of collagen synthesis, and higher levels of this cytokine have been noted in keloid tissue compared to normal skin [35].

Usually, most T cells are CD4+ memory T cells, while in keloids, there is a significantly higher proportion of CD8+ resident memory T cells (TRM) that are known to trigger an exaggerated inflammatory response to stimuli. Keloid memory T cells are less adept at producing TNF-α and more prone to generating IFN-γ resulting in exuberant but dysregulated T cell responses in keloids.

Within this pro-inflammatory state, increased intralesional and perilesional mast cells can be observed, both perivascularly and within abnormal collagen bundles, which can release several pro-angiogenic factors, including vascular endothelial growth factor (VEGF), fibroblast growth factor-2, platelet-derived growth factor (PDGF), and IL-6 [36,37]. In particular, VEGF-A represents a central driver for angiogenesis, which is increased in keloids. It is a main factor for regulating endothelial cell proliferation, migration, and vascular permeability. VEGF-A seems to interact with receptors (VEGFR1, VEGFR2) and it influences downstream pathways (MAPK/ERK, PI3K/AKT) [38].

TGF-β1 has a wide range of cellular sources, including fibroblasts, monocytes, T cells, and platelets. As a key regulator of fibrogenesis, it plays a crucial role in various cutaneous and solid organ fibrotic disorders, as well as in tumorigenesis through the activation of C-MYC.

Its action is expressed through the canonical TGF-β/Smad pathway: the binding of TGF-β to the serine/threonine kinase receptors leads to their phosphorylation allowing the Smad complex to translocate to the nucleus, where it regulates the transcription of target genes involved in ECM production and fibrosis, including genes encoding for collagen and connective tissue growth factors.

In keloids, TGF-β signaling is enhanced and fibroblasts become considerably sensitive to TGF-β, showing resistance to apoptosis and increased cell rigidity through the expression of smooth muscle actin (SMA).

IL-6 is pivotal to the transition from acute to chronic inflammation through the initiation of a profibrotic state by inducing pro-inflammatory cytokines (IL-1β and TNF-α) in monocytes. It is a strong activator of the extracellular signal-regulated kinase (ERK) 1/2–mitogen-activated protein kinase (MAPK) pathway and the JAK/STAT pathway, each of which has been implicated in keloid ECM gene expression and collagen synthesis. In particular, STAT3 activity is correlated with fibroblast proliferation and migration, as well as collagen deposition, mainly due to dysregulated secretion of cytokines resulting from altered epithelial–mesenchymal interactions

Moreover, another pathway involved in regulating fibroblast proliferation, migration, and differentiation into myofibroblasts is the phosphoinositide 3-kinase (PI3K)/AKT signaling pathway [36,37].

Recently, it has been demonstrated that microRNAs (miRNAs) and long non-coding RNAs (lncRNAs) are capable of modulating TGF-β/Smad signaling. A genome-wide transcriptome analysis has been performed to identify lncRNAs that are aberrantly expressed in keloid tissues. lnc-*LSP1P5*, a known lncRNA, resulted more elevated in the nuclei of fibroblasts in keloids, and it seems to silence CEBPA, which is an antifibrotic factor [39].

Interestingly, Schwann cells are a new category of cells recently discovered to be involved in the pathogenesis of keloids. In normal tissue, they are associated with axons, and they contribute to myelination and neuron development. In contrast, in keloids, they are associated with cell proliferation expressing genes linked to the regulation of inflammatory response [40]. In keloids, their number is markedly increased, and they have a profibrotic phenotype contributing to enhance ECM production. It may be hypothesized that this subgroup of Schwann cells with a different genetic expression can arise from a common initial damage of the tissue [41].

### 2.3. Etiology

Genetic factors contribute significantly to the pathogenesis of keloids. The inheritance pattern of keloid formation appears to be autosomal-dominant with incomplete penetrance, although environmental triggers (such as skin injury) are often necessary for their development [42]. Studies have shown that specific gene mutations in the ECM (extracellular matrix) and TGF-β signaling pathways may predispose individuals to the formation of keloids and that there is a higher risk of keloids in family members of affected individuals [27,43].

Keloids have been documented in association with certain congenital disorders in multiple reports in the literature. Among them, Rubinstein-Taybi syndrome is characterized by broad thumbs and big toes, a characteristic face, growth retardation, and intellectual disability of variable degrees. Bethlem myopathy, a collagen VI-related myopathy, presents early onset muscle weakness, proximal joint contractures, and distal joint laxity [44]. Goeminne syndrome is characterized by torticollis, cryptorchidism, renal dysplasia, and multiple nevi [43]. Dubowitz syndrome typically presents intrauterine growth retardation, low neonatal weight, short stature, and characteristic facies [12]. Noonan syndrome is a genetic multisystem disorder characterized by distinctive facial features, developmental delay, learning difficulties, short stature, congenital heart disease, renal anomalies, lymphatic malformations, and bleeding difficulties [45]. Conjunctiva–corneal dystrophy was described in 1998 and involves hereditary ocular dystrophic disease associated with keloid formation [13].

Some medical conditions, such as atopic conditions and asthma, are also known to be associated with multiple keloids, probably because they act on the same molecular pathways that lead to their development [26,27].

For example, the relationship between keloids and other disorders marked by the Th2 response, which is connected to wound healing, has been the subject of numerous studies, and in some cases, a positive association with atopic dermatitis was found. The IL-4Ra/STAT6 signaling pathway, a TGF-b-independent profibrotic mechanism, is activated when IL-4 and IL-13 bind to their respective receptors.

Another cell mediator that plays a pivotal role in various cutaneous and solid organ fibrotic disorders, including keloids, is TGF-b1. It induces smooth muscle actin (SMA) production in keloid fibroblasts leading to increased cell stiffness, which is a characteristic of both keloids and scleroderma [36].

In the end, there is evidence that some medications can predispose to keloid formation.

One of the most frequently reported drugs is isotretinoin, usually administered for acne treatment. Isotretinoin is a retinol derivative of vitamin A with effects on collagen formation, which can, partially, explain the occurrence of keloids in some patients [46]. In a case report, a 17-year-old girl diagnosed with a severe form of acne and treated for several months with isotretinoin 20 mg/day for 4 months had a marked improvement in the acne, but keloids were observed on the sternal area and shoulders. Similarly, another case of asthma was described, where keloid formation started after 8 weeks of treatment with isotretinoin 40 mg/day; keloids were localized on the trunk and persisted for years [23].

Also, letrozole, an aromatase inhibitor, has been reported to be associated with the development of keloid eruption, probably through a hormonally mediated pathway that promotes keloidogenesis [7,47].

### 2.4. Clinical Presentation and Symptoms

Keloids can form in various anatomical regions, but some skin areas are more commonly affected. According to a systematic review, the ear is the most common site for keloids, accounting for 40% of cases, followed by the chest (25%) and shoulders (20%) [31]. Interestingly, studies indicate that males tend to develop keloids more frequently on the chest and shoulders, while females often present with keloids on the earlobes and breast areas.

These lesions can be asymptomatic, but in most of cases, patients experience itching, pain, limited range of motion, and emotional and psychological distress due to the unpleasant esthetics of the scars [44].

Clinically, keloids present as firm, rubber-like, and raised lesions that are often red or purple in color initially, eventually becoming lighter and more fibrous over time. These lesions often grow slowly and progressively over several months or even years.

Patients with keloids may experience symptoms such as itching, pain, or tenderness, especially if the keloid is in areas such as the shoulders or chest. In some cases, keloids may cause functional impairment, particularly if they develop near joints or in areas with frequent movement. For example, keloids that form on the chest or back may limit the range of motion due to fibrosis, while those on the earlobes can cause esthetic concerns, particularly when they become large and unsightly [45].

While most keloids remain isolated lesions, some patients may develop multiple keloids, a condition known as generalized keloid disorder. This condition can be debilitating, affecting the quality of life due to both physical and psychological concerns, particularly in socially sensitive areas such as the face and neck or genital areas.

In our experience, two patients with presented keloids in the genital region at our outpatient clinic presented.

**Figure 1:**
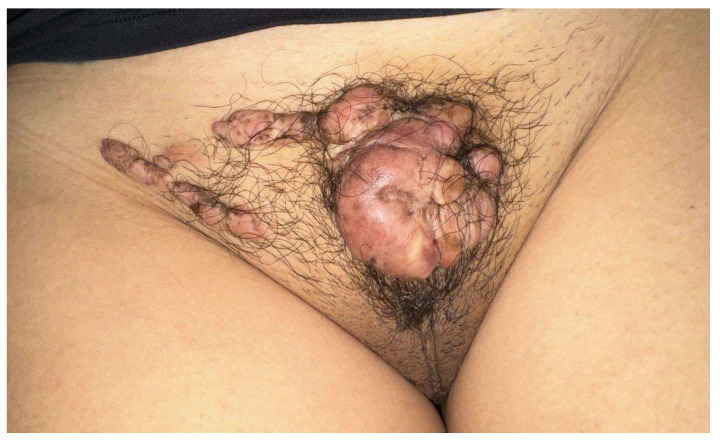
Multiple keloid lesions on the mons veneris.

The first case was a 15-year-old patient with multiple keloid lesions on the mons veneris; the keloids had arisen in the absence of areas of trauma related to pubic hair removal or practices such as tattoos at the age of 12. These keloids had increased in volume and number over time. The girl had undertaken her medical visits only three years after the appearance of the first lesions due to shame and fear linked to this pathology (Figure 1).

**Figure 2:**
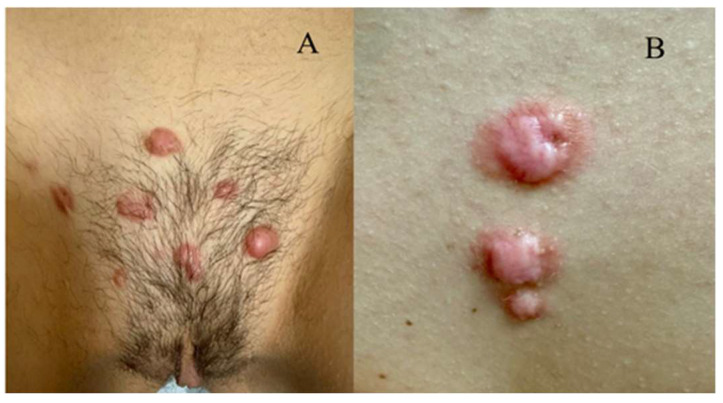
(**A**) Keloids on the mons veneris; (**B**) keloids in the intermammary sulcus.

The second case was a 12-year-old patient, with the appearance of multiple primary keloid lesions on the mons veneres and in the intermammary sulcus 2 years ago (Figure 2A,B).

### 2.5. Diagnosis

The diagnosis of keloids is primarily clinical, based on the history of lesion development, physical examination, and exclusion of other dermatological conditions. Key diagnostic features include a history of the lesion growing progressively beyond the initial wound margins, typically without a known injury or surgery. Dermoscopy may help in distinguishing keloids from other similar conditions, such as hypertrophic scars or dermatofibromas, by identifying pathognomonic characteristics like the absence of follicular structures and the presence of a dense vascular network [48].

In cases where the diagnosis is unclear, histopathological examination can confirm the suspect. Keloid tissue exhibits an overabundance of collagen fibers, typically organized in a haphazard manner, unlike the organized collagen seen in normal skin. A prominent characteristic of keloid tissue is the presence of dense, thick bundles of type I collagen, without a well-defined reticular dermis [49].

Despite clinical and histological diagnostic methods, keloids are often challenging to recognize in the early stages when they may resemble hypertrophic scars or other fibrous lesions. It is essential to differentiate these conditions to ensure accurate treatment strategies.

The differential diagnosis of keloid includes the following [27]:

- Hypertrophic scar: overgrowth of fibrous tissue that does not extend beyond territory of original injury. Size peaks within 12 months of initial injury and often regresses over time.
- Dermatofibroma: benign overgrowth of fibrous tissue forming single or multiple nodules, typically small (<1 cm), with color that varies from purple to yellow. It can be either painless or tender and rarely regresses.
- Dermatofibrosarcoma protuberans: dermal tumor that often begins as an asymptomatic papule and slowly grows, invading deeper structures and leading to the formation of nodules and ulcerations. It is most commonly found on the trunk, and color varies from flesh to reddish brown [50].
- Cutaneous sarcoidosis: noncaseating granuloma. Lesions are often raised and nodular, but they may vary in presentation.

## 3. Clinical Treatment

The treatment of keloids remains a clinical challenge, as there is no universally effective therapeutic approach, with all of them presenting low efficacy and side effects. Multiple strategies have been employed; however, the recurrence rate of keloids remains high, with up to 50% of cases returning after treatment [51].

A non-invasive therapy approach is silicone dressings (silicone gel sheets, silicone gels), which act by hydrating and obstructing the skin rather than directly preventing scarring. The hydration of the stratum corneum and the cytokine-mediated regulation of cell signaling between fibroblasts and keratinocytes are the mechanisms of action of silicone dressings. When compared to no treatment, silicone gel sheets demonstrated statistically significant improvements in scar color and thickness [37].

The most common medical approach is represented by the intralesional injection of corticosteroids, such as triamcinolone acetonide (TAC), which perform their action through different mechanisms. First, they decrease inflammation by inhibiting leukocyte and monocyte migration and phagocytosis. Second, they are potent vasoconstrictors, thus reducing the amount of oxygen and nutrients that reach the wound bed. Third, they have an antimitotic effect that suppresses collagen and glycosaminoglycan synthesis, reduces fibroblast proliferation, and augments collagen degradation [52].

Studies suggest that corticosteroid therapy has an efficacy rate of approximately 50–70% in improving keloid appearance, though the results can be variable depending on the size and location of the lesion [52]. The mechanism is based on the suppression of dermal inflammation, reduction in oxygen delivery to the wound bed via vasoconstriction, and antimitotic activity in keratinocytes and fibroblasts [53].

Nonetheless, TCA intralesional injections are commonly associated with side effects such as subcutaneous atrophy, telangiectasia, or developing recurrence. Research has shown that the concomitant use of platelet-rich plasma (PRP) could improve wound healing with lower keloid recurrence. In fact, PRP decreases inflammation and collagen deposition, supporting the deposition of more organized collagen structures in injured tissue giving a better cosmetic result and reducing symptoms.

Improvement was also noted with PRP injection in the surgical site after the excision of keloids [54].

Surgical removal of keloids is another option; however, this approach carries a high risk of recurrence, particularly when it is not followed by adjuvant treatments. The recurrence rate of surgery alone can be as high as 50–80% [51]. For this reason, excision is commonly followed by PRP, radiation therapy, intralesional therapy (e.g., TAG), cryotherapy, pression therapy, or silicone topical therapy, making it more effective.

Laser devices, ablative or not, such as YAG laser, pulsed dye laser (PDL), fractional CO_2_ laser, and KTP (Potassium-Titanyl-Phosphate) laser, in combination with TAC or alone, have been shown to be effective in treating keloids. A systematic review by de las Alas et al. demonstrated that PDL may lessen erythema when paired with TAC, even if it might not be as successful as FCO_2_ plus 5-FU in lowering scar thickness and enhancing pliability. Fractional CO_2_ laser, which targets collagen remodeling, has also been shown to improve keloid appearance in some studies.

Moreover, a systematic review by Mirza et al. described the possibility of using ablative, non-ablative, or a combination of laser therapies during systemic isotretinoin treatment with almost no side effects [55].

Cryotherapy is another option, often used in conjunction with corticosteroid injections. It works by inducing tissue necrosis and collagen remodeling, but its efficacy is lower compared to corticosteroid injections, with a success rate of about 50% [45].

In recent years, 5-aminolevulinic acid-based (5-ALA) photodynamic therapy (PDT) has gained growing interest in this field. The therapeutic procedure consists of three essential elements: a photosensitizer (5-ALA), laser irradiation, and the production of reactive oxygen species (ROS). 5-ALA, a precursor of heme biosynthesis, when permeated in mitochondria, could be enzymatically converted into protoporphyrin IX, which in turn interacts with laser to produce ROS that ultimately trigger cell death [56].

In addition, there is a group of drugs, used for other pathologies, whose mechanism of action is exploited for the treatment of keloids. ACE-inhibitors, oral or topical, reduce fibroblast proliferation. An antiallergic agent, tranilast, suppresses type I allergic reactions by inhibiting the release of chemical mediators such as histamine and leukotrienes, and it also inhibits the production of collagen and angiogenic and inflammatory factors. Calcium antagonists, such as verapamil and nifedipine, can suppress extracellular matrix protein synthesis.

Immunosuppressors like tacrolimus, which inhibits collagen proliferation, migration, and production enhanced by TGF-β1 and imiquimod, which induces IFN-α, along with monoclonal antibodies (Dupilumab, Anti-TGF-β1 Antibody, Anti-VEGF-A Antibody) targeting specific cytokines involved in wound healing could help prevent/treat keloids.

Some chemotherapeutics (Bleomycin, 5-Fluorouracil, Mitomycin C, Paclitaxel, Tamoxifen) have been proposed as effective modalities for scar treatment and scar prevention in addition to steroid injection or after surgery, because these drugs target the fibroblasts in scar tissue, and induce apoptosis or modulate protein production.

For esthetics purposes, intralesional injections of collagenase or hyaluronidase can stimulate angiogenesis and activate mesenchymal stem cells, which improve the function as well as the cosmetic appearance of scar tissues.

Fat-Soluble Vitamins (Vitamin A, Vitamin D3, Vitamin E) can be utilized for their antioxidant and anti-inflammatory properties to speed up wound healing [57].

Punch excision therapy for keloids involves multiple drill holes at a distance of 0.5 mm from each other. It reduces the mechanical forces within the scar, which aids the wound healing. A controlled trial compared punch excision combined with intralesional steroid injection with only intralesional steroid injection, showing that the first group had better outcomes than the second one. In addition, punch biopsy resulted as less traumatic and simpler than nuclear excision [58].

In recent years, newer therapies targeting specific molecular pathways involved in collagen synthesis have been promising, but these treatments are still in the experimental phase. Among them, biologics targeting specific immune pathways, such as TGF-β/Smad and STAT3, and small-molecule inhibitors, for the reduction of fibroblast proliferation and ECM production are emerging as promising therapeutic options for the treatment of keloid scars. Various inhibitors targeting the JAK-STAT pathway (e.g., Ruxolitinib) have also shown potential therapeutic effects on keloids. Additionally, small-molecule inhibitors targeting the MAPK and PI3K/AKT pathways (e.g., Sorafenib) are under investigation for their potential to reduce keloid formation.

Another option is RNA therapy, which uses small interfering RNA (siRNA) to modulate gene expression involved in fibrosis. It offers several advantages: siRNAs have a high specificity and can target a large number of genes, even ones that were once thought to be “undruggable” and their high specificity can potentially reduce side effects compared to traditional small molecules.

Eventually, mesenchymal stem cell (MSC) therapy has gained significant interest as a novel approach for treating keloid scars due to its anti-inflammatory and antifibrotic properties [36].

It is important to underline that the outcomes of the therapies cited above are heterogenous, and it is difficult to compare the different studies. Patients have different family histories, keloid localization, skin types, sizes, and numbers of keloids, and all of these factors can influence treatment response [53].

In conclusion, to increase the clinical efficacy of pharmacotherapy in treating keloids, it is crucial to select the treatment method depending on the patient’s symptoms, the site and size of the lesion, and mode of administration. Combination pharmacotherapy is much more effective in treating and/or preventing the recurrence of keloids, compared to mono-pharmacotherapy, due to its multiple mechanisms of action on the affected sites simultaneously.

## 4. Conclusions

This review has some limitations, such as the inclusion of studies that have small sample sizes, lack randomized control trials, and employed a short follow-up of patients enrolled in the considered paper; these may influence the results.

Keloids represent a significant clinical challenge due to their multifactorial nature and complex pathogenesis. Keloids are a rare, yet described, medical condition. Although these lesions appear without a specific recalled injury, there are some known causes that, along with some clinical and histopathological signs, can help to identify and distinguish them from other similar lesions, such as hypertrophic scars or provoked keloids.

Additionally, current treatment modalities offer varying degrees of success, but new advances in targeted therapies and personalized medicine offer hope for better management in the future.

The keloid microenvironment exhibits a robust inflammatory response to both mechanical and non-mechanical stimuli, leading to a complex interaction among various hyperactivated immune components, ultimately resulting in a profibrotic cytokine profile and signaling pathways. Based on this knowledge, further research into the molecular mechanisms underlying keloid formation, along with the development of more effective targeted treatments, remains crucial in improving patient outcomes.

## Data Availability

The data that support the findings of this study are available from the corresponding author, [M.D.], upon reasonable request.
